# Insight into HIV of IFN-Induced Myxovirus Resistance 2 (MX2) Expressed by Traditional Chinese Medicine

**DOI:** 10.1155/2014/871576

**Published:** 2014-06-18

**Authors:** Tzu-Chieh Hung, Wen-Yuan Lee, Kuen-Bao Chen, Yueh-Chiu Chan, Calvin Yu-Chian Chen

**Affiliations:** ^1^Department of Biomedical Informatics, Asia University, Taichung 41354, Taiwan; ^2^School of Medicine, College of Medicine, China Medical University, Taichung 40402, Taiwan; ^3^Department of Neurosurgery, China Medical University Hospital, No. 2 Yude Road, North District, Taichung 40447, Taiwan; ^4^Department of Anesthesiology, China Medical University Hospital, Taichung 40447, Taiwan; ^5^Research Center for Chinese Medicine & Acupuncture, China Medical University, Taichung 40402, Taiwan; ^6^Human Genetic Center, Department of Medical Research, China Medical University Hospital, Taichung 40447, Taiwan

## Abstract

Recently, an important topic of the acquired immunodeficiency syndrome (AIDS) had been published in 2013. In this report, the expression of the IFN-induced myxovirus resistance 2 (MX2) had been defined the function to kill the human immunodeficiency virus (HIV). The screening from the Traditional Chinese Medicine (TCM) database by simulating molecular docking and molecular dynamics could select candidate compounds, which may express MX2 against HIV. Saussureamine C, Crotalaburnine, and Precatorine are selected based on the highest docking score and other TCM compounds. The data from molecular dynamics are helpful in the analysis and detection of protein-ligand interactions. According to the docking poses, hydrophobic interactions, and hydrogen bond with structure variations, this research could assess the interaction between protein and ligand interaction. In addition to the detection of TCM compound efficacy, we suggest that Saussureamine C is better than the others in protein-ligand interaction and the structural variation to express MX2.

## 1. Introduction

The human immunodeficiency virus (HIV) is a retrovirus that causes humans to have the acquired immunodeficiency syndrome disease (AIDS) [1–4]. In this disease, immune system of patient is compromised by the virus, which then allows opportunistic infections, and cancers, to flourish. In the statistics, the main reasons for HIV virus transmission are unprotected sexual intercourse [5, 6], contaminated medical equipment [7, 8], vertical infection (pregnancy, delivery, or breastfeeding) [9, 10], and bodily fluids.

Since the first case in 1981, AIDS has caused nearly thirty-six million deaths and there were still seventy-five million carriers, as reported by UNAIDS (http://www.unaids.org/en/resources/campaigns/globalreport2013/factsheet/). There are still no vaccines or drugs available to kill the virus in clinical treatment; therefore, the highly active antiretroviral therapy (HAART) becomes the standard care for patients with advanced infection [11]. HARRT uses a complex of transcription inhibitors to decrease the patient's total numbers of HIV virus, but it is expensive and inconvenient medication.

Recent research has pointed out that HIV of IFN-induced myxovirus resistance 2 (MX2) is an important gene against AIDS [12]. The MX is a resistant system to kill virus by overexpression of the IFN-*α* which imposes an early block to HIV-1 reverse transcription. In human, there are two known kinds of MX system named MX1 and MX2. The MX1 is known as the function against influenza and the MX2 has defined to resist HIV. Thus, a lot IFN be produced from MX2 expressed could be a better treatment for HIV patients [13].

Computer-Aided Drug Design (CADD), which is an* in silico* simulation technique to screen for compounds by the structure and the biological activity of drug candidates, has the advantages of both greater speed and lower cost than traditional drug design. There are two major application areas named structure-based drug design and ligand-based drug design of CADD [14–19]. In this research, we used CADD to investigate the ligand efficacy based on structure-based drug design and molecular dynamics.

The personalized medicine and biomedicine [20] become a well-known knowledge which could analyze the disease associated with the mutation [21], pathway, and even discuss the cause for special disease [22]. Traditional Chinese medicine (TCM) is a kind of personalized medicine which is an important medical culture in Asia. The TCM Database@Taiwan (http://tcm.cmu.edu.tw/) [23] is the largest traditional Chinese medicine database in the world built in 2011. This TCM database contains 2D chemical structures, 3D chemical structures, bioactivity, and molecular information for over 61,000 compounds of TCM herbs. From 2011 to 2014, there have been successful discoveries of novel lead compounds from the TCM Database@Taiwan for cancer treatment [24–27], pain relief [15], and antivirals [28–32]. With the assistance of the application system of the website [33] and the cloud computing platform [34], the TCM Database@Taiwan could be valuable for TCM application and drug design.

In this study, we screen the TCM Database@Taiwan to select a possible lead compound against HIV. We use biocomputational technology as molecular docking screening to select ligands. Finally, we apply molecular dynamics (MD) simulation to discuss the protein-ligand interactions that may contribute to the evaluation of the effect of MX2 expression.

## 2. Materials and Methods

### 2.1. Data Set

Accelrys Discovery Studio 2.5 (DS 2.5) was used for the molecular simulations. A total of 61,000 TCM compounds were downloaded from the TCM database (http://tcm.cmu.edu.tw/). The MX2 sequence was generated from UniProt (ID: P20592) and made the prediction of the 3D structure from Ittarsser wed server (http://zhanglab.ccmb.med.umich.edu/I-TASSER/) [35]. Then to detect correction for the residue of prediction MX2 structure, we submit the structure to rampage Ramachandran (http://mordred.bioc.cam.ac.uk/~rapper/rampage.php).

### 2.2. Disorder Protein Detection

Because the disorder plays an important role in drug design, we submit protein structure and docking site to predict the disorder region by the Database of Protein Disorder (DisProt: http://www.disprot.org/) [36]. As a result of the prediction, we can decide the character of the docking site and the efficacy of the drug [17, 37].

The docking site designed nearby the important amino acids based on UniProt was reported. Based on a comparison of the disorder regions and the docking sites, we could assess the protein-ligand interaction and drug efficacy.

### 2.3. Molecular Docking

The docking simulation used the LigandFit [38] module to dock the TCM compounds to MX2 in the force field of CHARMm [39]. LigandFit is a receptor-rigid docking algorithm program in Discovery Studio 2.5 (DS 2.5). The docking site of MX2 was identified by the nucleotide binding region which was recorded as the relation with GTP function. After docking, the top three docking scores of the compounds were selected and then analyzed for hydrophobic interactions by Ligplus [40, 41].

### 2.4. Molecular Dynamics Simulation

These ligands must be reprepared based on the reference force field [42] of GROMACS 4.5.5 [43] by using SwissParam (http://swissparam.ch/) [44] before applying MD simulation. A simulation box is performed for complex of the protein with candidate compound. The cubic box with a minimum distance of 1.2 Å from the complex was solvated based on the TIP3P water model. The TIP3P water model supports sodium and chloride ion for simulation to neutralize complex charges. The complex takes the minimization with the Steepest Descent method for 5,000 steps. The last structure with the minimum energy was transferred to MD simulation. Electrostatic interactions were calculated based on the Particle-Mesh Ewald (PME) method [45] with each time step being 2 fs and the numbers of steps were 2,500,000 times. The Berendsen weak thermal coupling method for the equilibration was under the 100 ps constant temperature (PER ensemble). The total simulation time of MD was 5,000 ps. MD trajectories, RMSD, energy, H-bond, and eigenvector calculation of the complex were analyzed using a series of protocols in Gromacs.

## 3. Results and Discussion

### 3.1. The Detection of Disorder Protein

**Figure 1:**
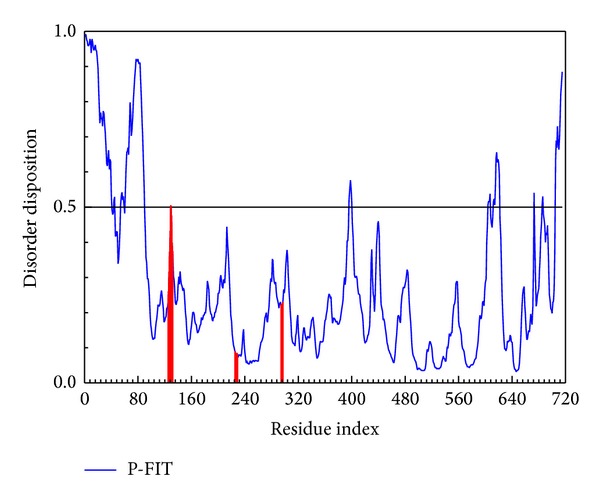
The disorder and binding site detection. The blue curve is the disorder disposition of each amino acid, and the red lines are the residues of docking region.

The disorder protein is defined as unstructured protein. While the docking site consists of a disorder region, the drug docks to protein hardly and the complex will stabilize with difficultly. The cited references [17, 37] denote that the ligand docks into the disorder region may have weaker side effect than the widespread domain. Therefore, the disorder region should be defined as a hard work for drug design and not a bad docking site for selection. The disorder regions of MX2 are defined as having a disposition of over 0.5 (Figure 1). This result presents the most residue of MX2 for docking site design that do not consist of disorder regions; thus the docking simulation is appropriate without disorder infection.

### 3.2. Prediction Protein Modeling Detection

**Figure 2:**
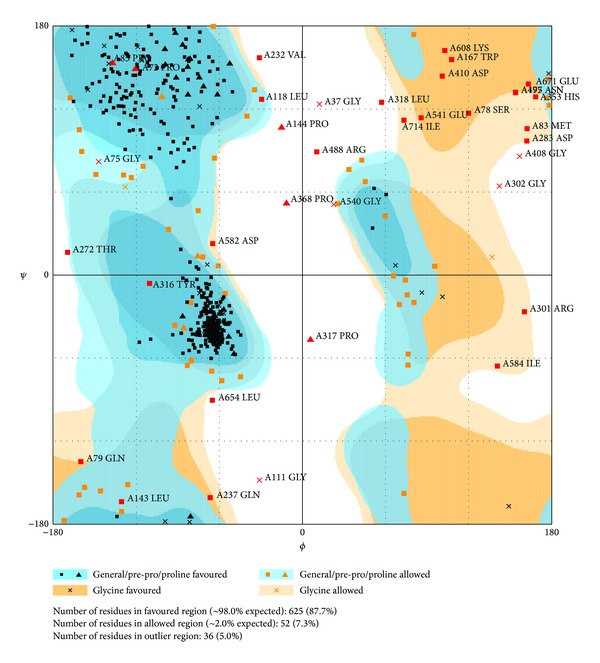
The prediction structure detection. RAMPAGE by Paul de Bakker and Simon Lovell is available at http://mordred.bioc.cam.ac.uk/~rapper/rampage.php [61].

The prediction model should detect the credibility of the structure. The rampage Ramachandran uses the structure of each residue to evaluate the unreasonable position that will make amino acids collide while the protein twists (Figure 2). In this result, the amino acids Gly37, Gly75, Gly111, Gly302, Gly408, Gly540, and Glu671 are defined as unreasonable position in MX2. Fortunately, these amino acids are not as important as acids and docking site; thus the prediction model is credible for simulation.

### 3.3. Molecular Docking

After molecular docking and ranking by docking score, the top three TCM compounds can be selected (Table 1). These TCM compounds are Saussureamine C, Crotalaburnine, and Precatorine extracted from the TCM herbs* Saussurea lappa *Clarke,* Crotalaria laburnifolia (*or* Crotalaria anagyroides), *and* Abrus precatorius*, respectively. The top compound, Saussureamine C, is found having antiulcer function [46] and its herb* Saussurea lappa *Clarke can prevent breast cancer [47], treat heart disease [48, 49], have antihepatotoxic activity [50], and express the cytotoxic T lymphocytes [51]. The second ranked compounds with the herb do not have reference about immunity. The third ranked herb* Abrus precatorius* had been defined the relation about Antiprotozoal [52, 53], antimicrobial [54–56], anti-inflammatory [57], apoptosis [58, 59], and immunotoxin [60]. Most of the reported literatures indicate that these compounds can have an effect on immunity; therefore we suggest that the selected compounds can have an influence on MX2.

**Figure 3:**
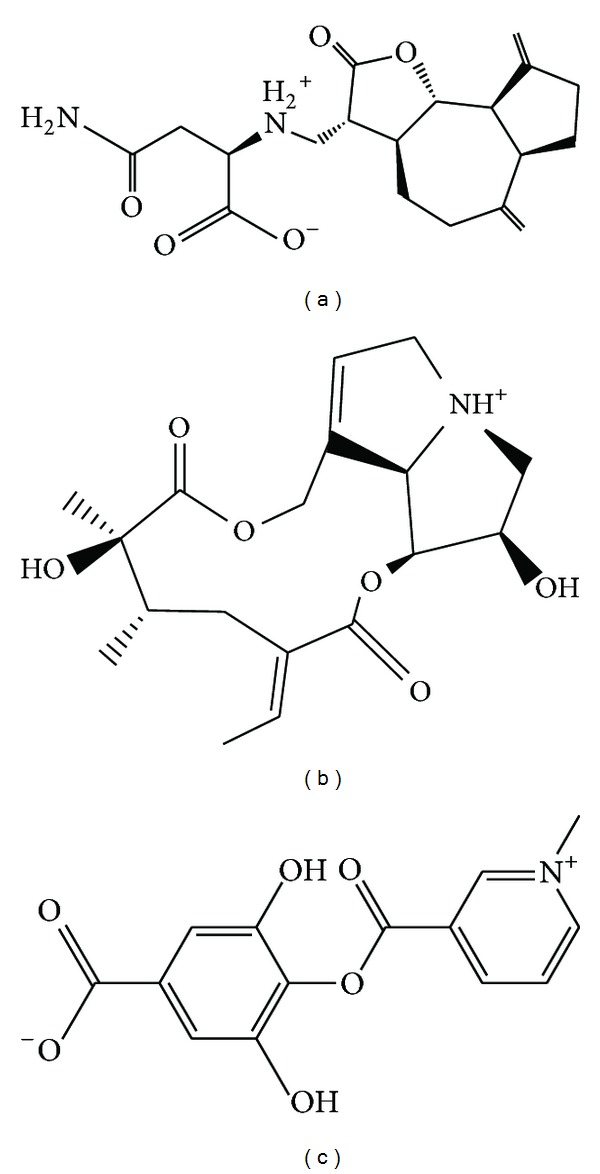
The structure of candidate TCM compounds. (a) Saussureamine C, (b) Crotalaburnine, and (c) Precatorine.

**Figure 4:**
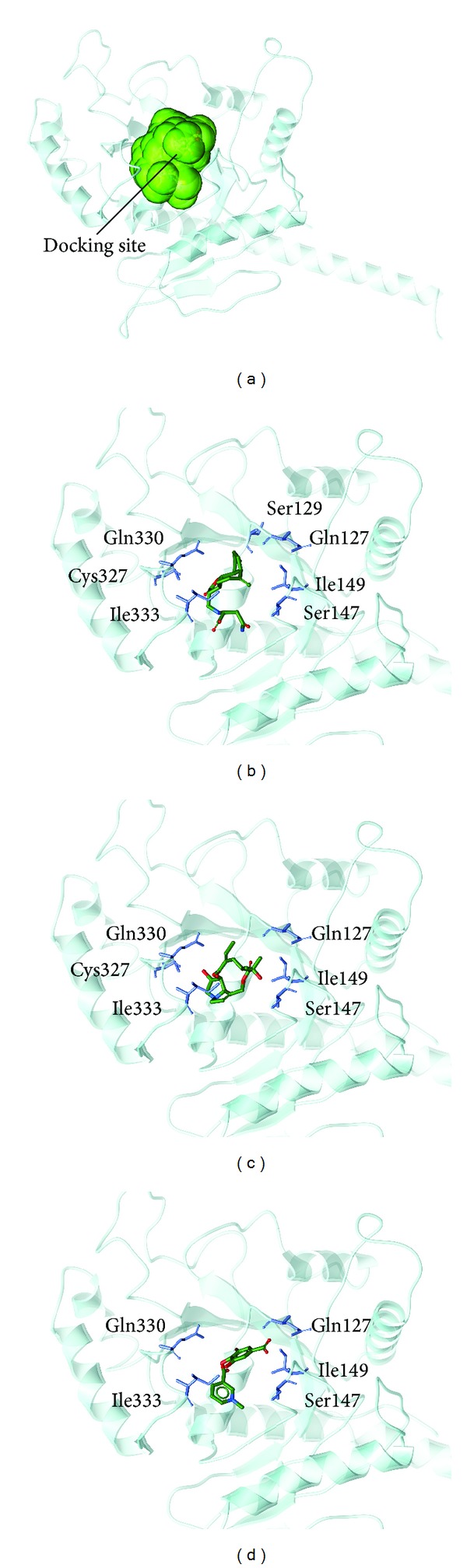
The docking poses of ligands. (a) The crystal structure of MX2 and the docking site, (b) Saussureamine C, (c) Crotalaburnine, and (d) Precatorine.

The structure of the candidate compounds screened from TCM database is displayed in Figure 3. The docking poses and the neighbor amino acid in binding site are presented in Figure 4. This result indicates that the residues Gln127, Ser147, Ile149, Gln330, and Ile333 have been interacted with all ligands. Thus, we could be suggested that these amino acids may play important roles for ligand to bind with MX2.

**Figure 5:**
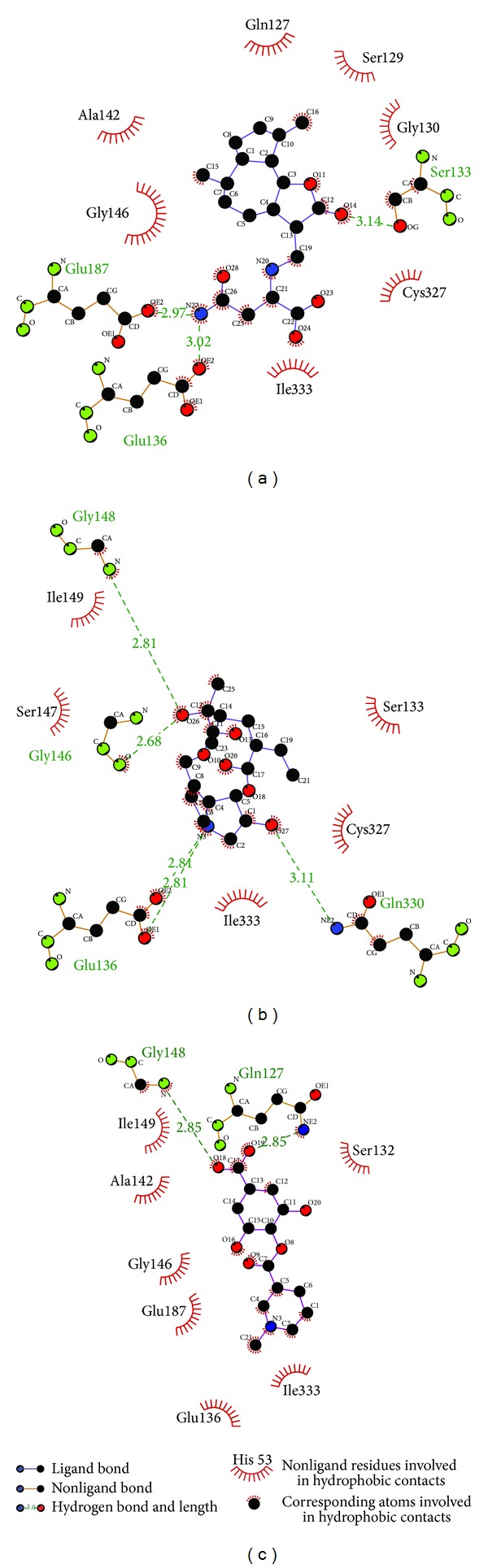
Ligplot illustrates the protein-ligand interactions. (a) Saussureamine C, (b) Crotalaburnine, and (c) Precatorine. The residue with deep red color indicates a high frequency in all ligand interactions.

The program Ligplus can analyze the possible H-bond and hydrophobic interaction (Figure 5). In this figure, the amino acids colored deep red are at high frequency while proteins have interactions with the ligands through hydrophobic interactions or hydrogen bonds. Thus these amino acids supply the possibility that these amino acids may have the function on protein-ligand interaction.

### 3.4. Molecular Dynamics Simulation

**Figure 6:**
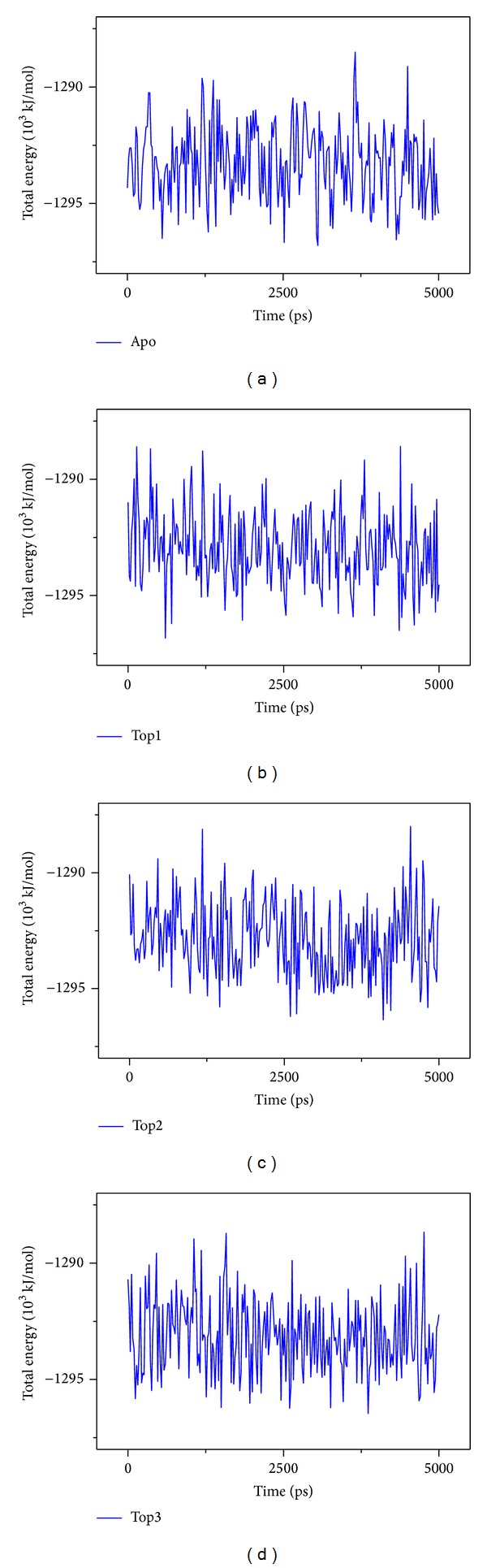
The total energy in MD simulation. Apo/unbound protein, top1 (Saussureamine C), top2 (Crotalaburnine), and top3 (Precatorine).

The RMSD and total energy of a complex during MD simulation were recorded (Figure 6). The total energy is in the range between −1295~−1285∗10^3^ kJ/mol and tends to be −1290∗10^3^ kJ/mol. The amplitude tends to be gentle and the energy is lower which indicates that the complex is more and more stable.

**Figure 7:**
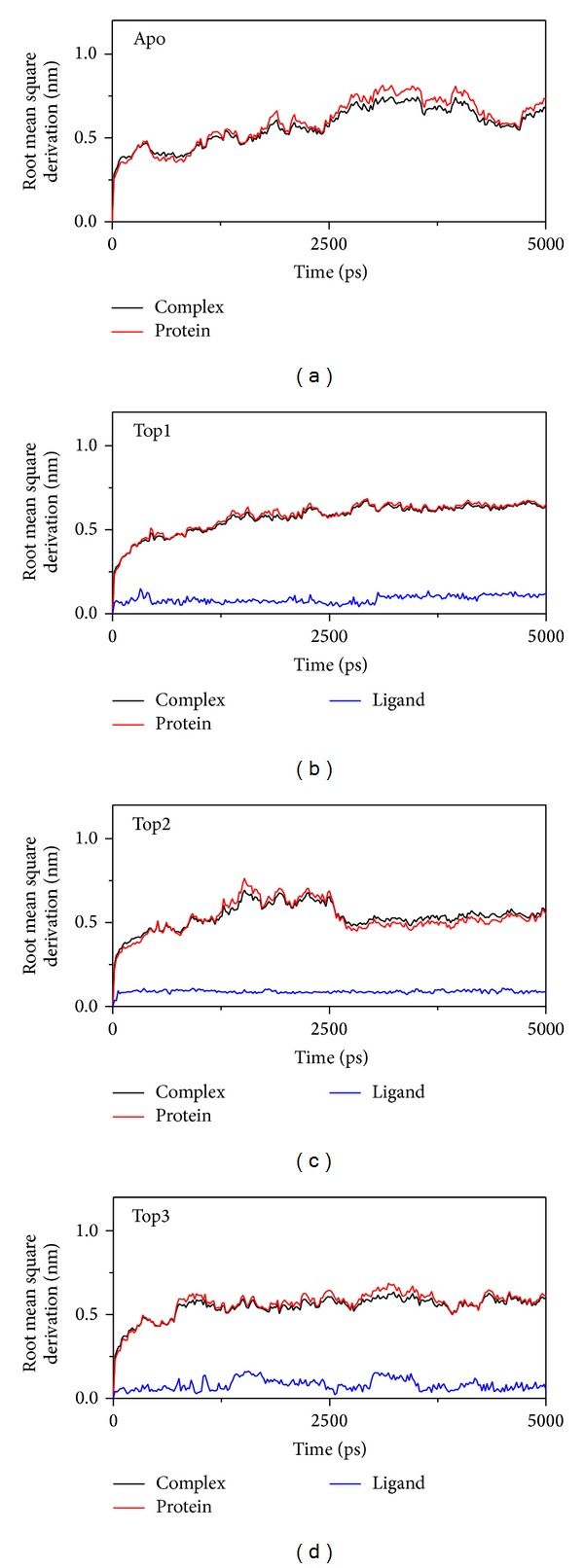
The RMSD in MD simulation. apo/unbound protein, top1 (Saussureamine C), top2 (Crotalaburnine), and top3 (Precatorine).

The root mean square derivation (RMSD) is the calculation of the root mean square for each atom in MD to describe the position variation focus on protein, ligand, and complex (Figure 7). In this figure, the RMSD variation of the complexes are lower than of Apo form (unbound protein), which presents that the conformation of MX2 with compounds will be more stable than unbound one.

**Figure 8:**
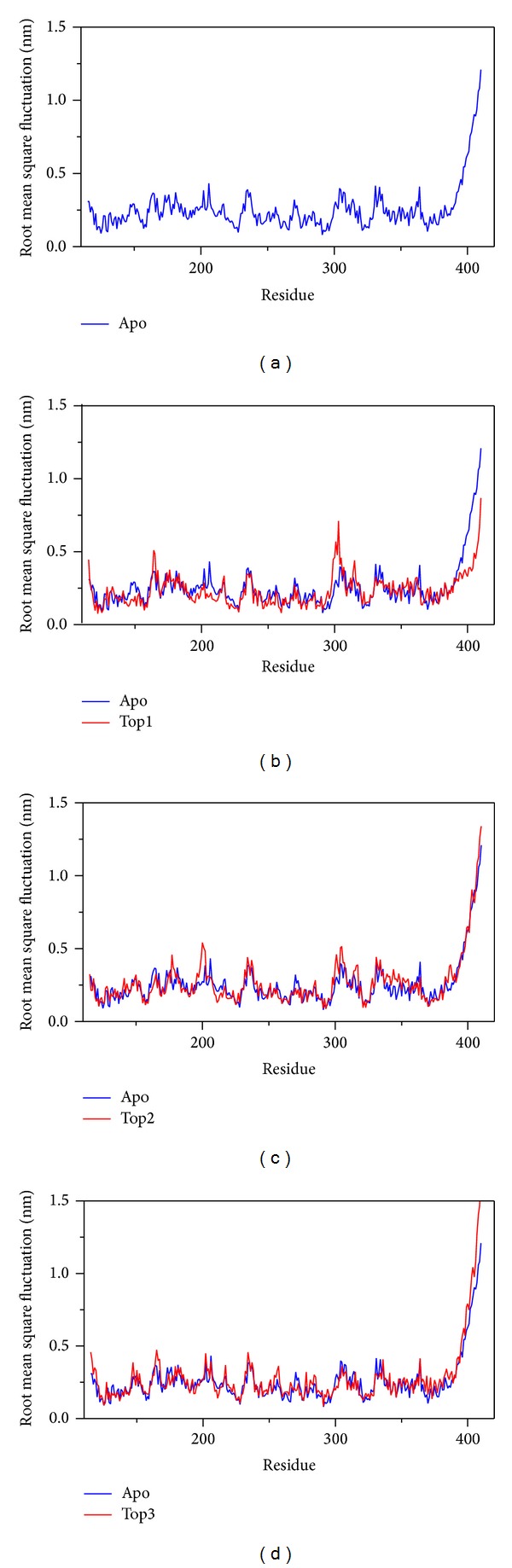
The RMSF compared with Apo/unbound in MD simulation. apo/unbound protein only, then with top1 (Saussureamine C), top2 (Crotalaburnine), and top3 (Precatorine).

The RMSF is the average of RMS for each residue position variation in the whole MD (Figure 8). To compare the RMSF between the protein in complex and in Apo form (unbound protein), we could find the effect of each ligand to similar residues in important region of protein. In Figure 8, the similar pick site confirms that the docking site is designed correctly and the top1 compound Saussureamine C has better effect on MX2 than others.

**Figure 9:**
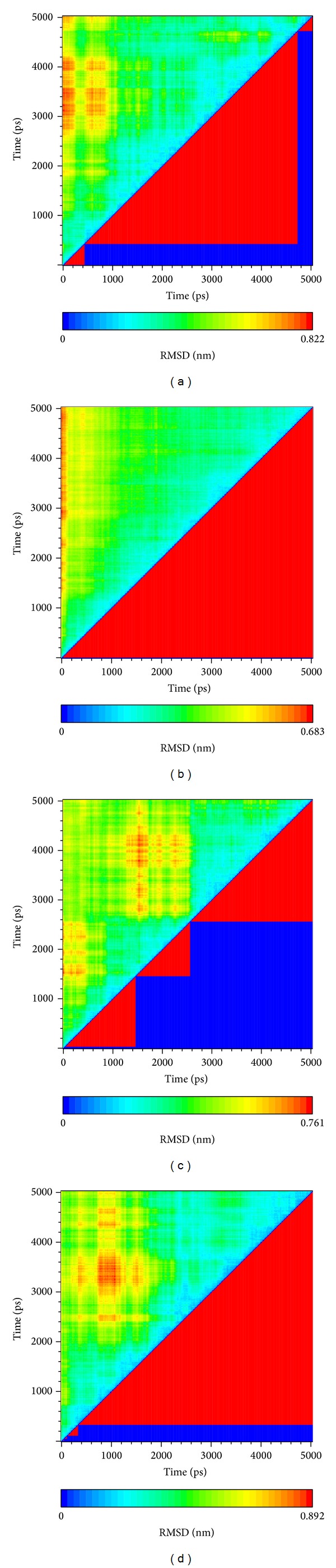
The clustering of the ligand-protein interaction. (a) apo, (b) Saussureamine C, (c) Crotalaburnine, and (d) Precatorine.

The clustering is a method to classify the MD trajectaries into several groups using RMSD variation (Figure 9). In this figure, the largest group of Apo form (unbound protein) is not the last group which may present this structure of Apo form is unstable in simulation. Thus, the clustering for complex means that the protein with ligand will be stable. Among these compounds, the Saussureamine C could make a balance quickly.

**Figure 10:**
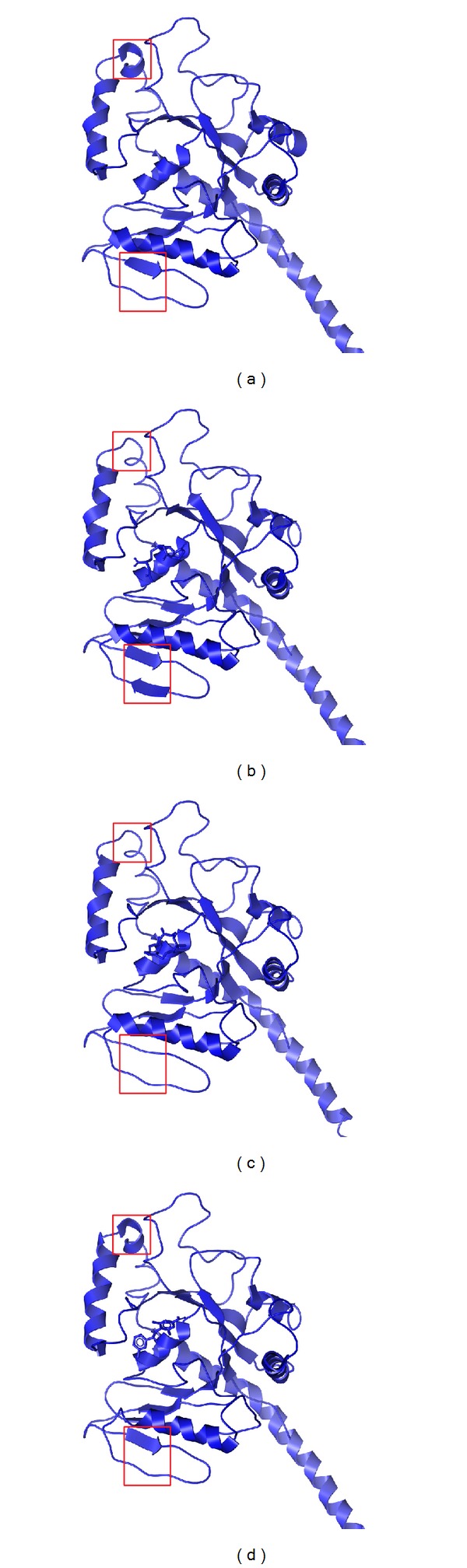
The structure variation before MD. (a) apo, (b) Saussureamine C, (c) Crotalaburnine, and (d) Precatorine. The site colored in red means the difference.

**Figure 11:**
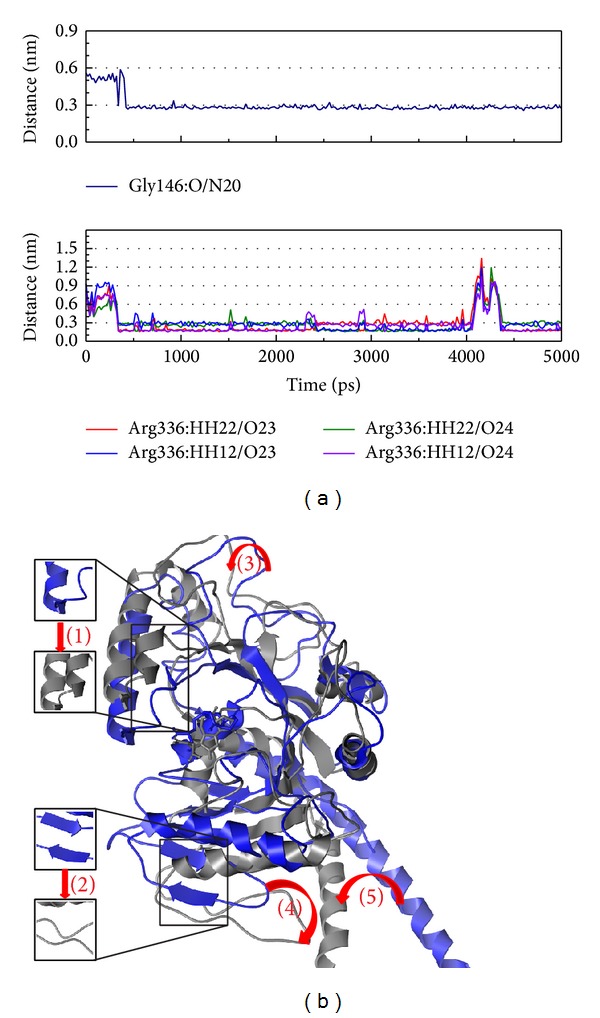
The variation of Saussureamine C and MX2 complex in MD simulation.(a) H-bond variation and (b) structure variation. The (1)–(5) red color indicates the difference through MD.

**Figure 12:**
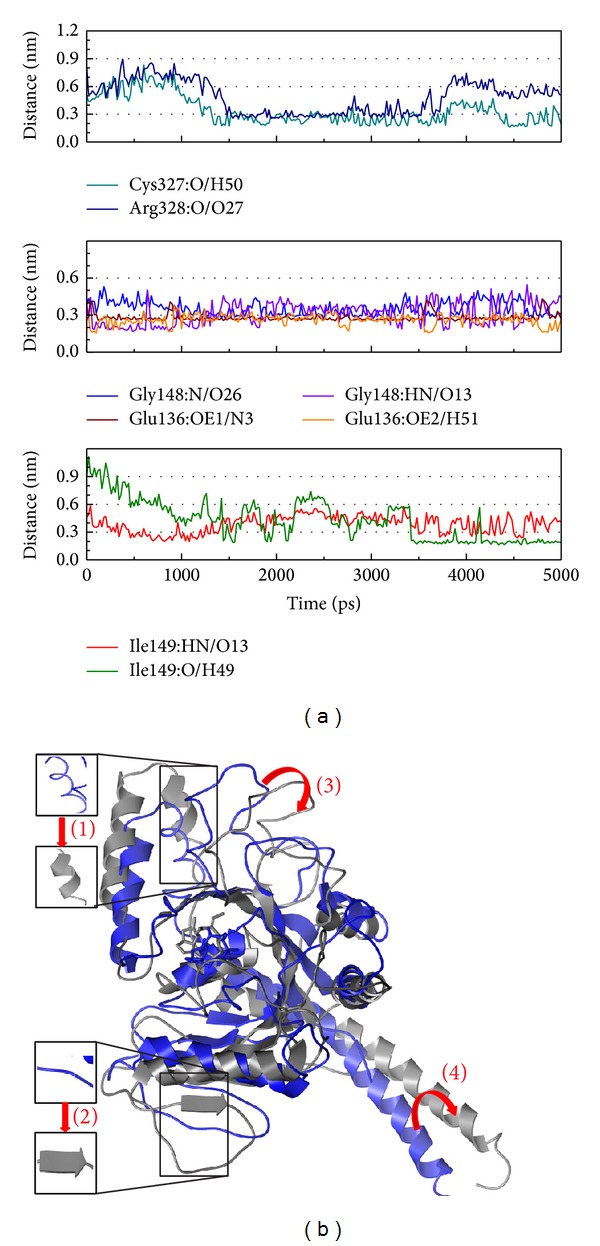
The variation of Crotalaburnine and MX2 complex in MD simulation.(a) H-bond variation and (b) structure variation. The (1)–(4) red color indicates the difference through MD.

**Figure 13:**
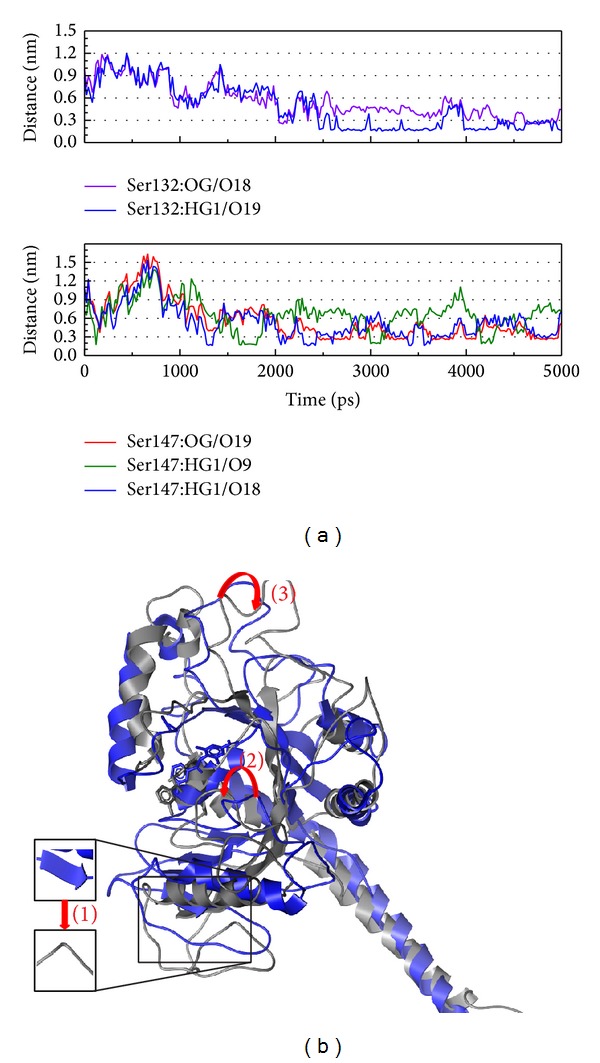
The variation of Precatorine and MX2 complex in MD simulation. (a) H-bond variation and (b) structure variation. The (1)–(3) red color indicates the difference through MD.

The structure variation could help in discussing the interaction and the function expressed from ligand effect; thus the analysis of structure variation is necessary (Figures 10 to 13). In Figure 10, the compound has made the protein variation after compound dock to protein. In this result, we found that the selected compounds could make strong influence on MX2, besides Precatorine.

In Figure 11(a), the Gly146 and Arg336 have high H-bond occupancy during the MD simulation. We suggest that this situation might make the compound close to docking site; then the compound could have more effect on functional domain.  Figure 11(b) presents the large composition variation of MX2 while interacting with Saussureamine C. We suggest Saussureamine C could have strong effect on MX2 in this situation.

The Crotalaburnine has a lot H-bond from different residues (Figure 12(a)) and in Figure 12(b), the Crotalaburnine is presented a strong effect for the variation of protein position and composition.

In Figure 13(a), the data recorded that Precatorine could become H-bond with Ser132 and Ser147 but the occupancy is less than others (it means that the frequency is less than other selected compounds). The protein variation could be found, especially around the docking site.

Based on the above discussion, we found that there is highly H-bond occupancy in protein-ligand interaction from different residues nearby docking site. In this situation, we suggest that the H-bond play an important role for the compounds to bind with MX2. It might be presented the binding affinity of ligands to MX2. For this reason, strong force interaction of H-bond make protein and ligand to be more stable. Then, the situation of high H-bond occupancy in MD simulation cause the complex with Saussureamine C tend to stable quickly. Finally, this research indicates that the interaction of compound with the residue in the binding site might active MX2 and simulate the immunity function.

## 4. Conclusion

Based on above discussion, we found that the top three TCM compounds Saussureamine C, Crotalaburnine, and Precatorine can have an effect on MX2 against the HIV alive. The residue around the docking site might use H-bond interaction to make antigen or compound simulate the immunity function through MX2 expressed. The structural variations indicate that all compounds can have an effect on immunity function, but Saussureamine C has the best effect on the activation of MX2.

## Figures and Tables

**Table 1:** Scoring functions of the top three compounds, and the expressors of MX2.

Compounds	Herbs	-PLP1	-PLP2	Dock Score
Saussureamine C	*Saussurea lappa *Clarke	46.53	41.62	154.496
Crotalaburnine	*Crotalaria laburnifolia* *or CrotaIaria anagyroides *	51.38	43.86	139.829
Precatorine	*Abrus precatorius* L.	29.18	27.57	139.744
